# Large Animal Models of Heart Failure With Reduced Ejection Fraction (HFrEF)

**DOI:** 10.3389/fcvm.2019.00117

**Published:** 2019-08-14

**Authors:** Andreas Spannbauer, Denise Traxler, Katrin Zlabinger, Alfred Gugerell, Johannes Winkler, Julia Mester-Tonczar, Dominika Lukovic, Claudia Müller, Martin Riesenhuber, Noemi Pavo, Mariann Gyöngyösi

**Affiliations:** Division of Cardiology, Department of Internal Medicine II, Medical University of Vienna, Vienna, Austria

**Keywords:** heart failure, heart failure with reduced ejection fraction, translational research, large animal models, porcine, ovine, canine, review

## Abstract

Heart failure with reduced ejection fraction (HFrEF) is defined by an ejection fraction (EF) below 40%. Many distinct disease processes culminate in HFrEF, among them acute and chronic ischemia, pressure overload, volume overload, cytotoxic medication, and arrhythmia. To study these different etiologies the development of accurate animal models is vital. While small animal models are generally cheaper, allow for larger sample sizes and offer a greater variety of transgenic models, they have important limitations in the context of HFrEF research. Small mammals have much higher heart rates and distinct ion channels. They also have much higher basal metabolic rates and their physiology in many ways does not reflect that of humans. The size of their organs also puts practical constraints on experiments. Therefore, large animal models have been developed to accurately simulate human HFrEF. This review aims to give a short overview of the currently established large animal models of HFrEF. The main animal models discussed are dogs, pigs, and sheep. Furthermore, multiple approaches for modeling the different etiologies of HF are discussed, namely models of acute and chronic ischemia, pressure overload, volume overload as well as cytotoxic, and tachycardic pacing approaches.

## Introduction

Heart failure (HF) is a complex clinical entity with multiple different etiologies. The primary characteristic of HF is reduced contractile force or inadequate filling. The main causes are ischemic injury as well as pressure or volume overload. Globally, more than 26 million patients are affected, with prevalence reaching more than 12% above 70 years of age (1, 2). HF is further categorized into three subgroups; (1) HF with reduced ejection fraction (HFrEF) with an ejection fraction (EF) below 40%; (2) HF with midrange EF (HFmrEF) with an EF of 40–50%; (3) HF with preserved EF (HFpEF) with EF >50%. The relative incidence of these three subgroups has been shifting in recent decades, toward declining HFrEF, and increasing HFpEF. According to a recent 30-year analysis of the Framingham Heart Study, newly diagnosed HF consists of 56.2% HFpEF, 31.1% HFrEF, and 12.8% HFmrEF (3). This shift is due to improvements in the management of acute myocardial infarction and better management of atherosclerotic risk factors, such as hyperlipidemia. However, it is important to note that while the relative proportion of HFrEF is decreasing, the absolute number of HFrEF patients is projected to increase over the coming decades.

### Importance of Large Animal Models of HFrEF

Animal models in general have been a cornerstone of basic and translational research. Among available models of human diseases, small mammals like mice, and rats are used in the vast majority. Their advantages include relatively low cost, and space requirements, which enable larger sample sizes, availability of many transgenic models, and short life-cycles, which allow the modeling of disease over an entire lifespan.

In comparison, large animal models are more labor-intensive, costly and they require larger, and more specialized facilities. Due to longer generation times, very few transgenic models exist, and new ones are difficult to establish. However, they have several significant advantages that make them an absolute necessity in basic and translational research.

There are several physiological differences between small mammals and humans that are relevant for cardiovascular research. Prominent examples are much faster heart rates in mice (300–840 beats per min) and rats (330–480 beats per min), along with drastically different action potential durations, a different basal metabolic rate, and oxygen consumption as well as phenotypic differences in their stem cells (4). Therefore, extrapolation of data from small animal models to humans is difficult. This is best showcased by a long list of therapies and molecular targets that seemed promising in rodents but then failed to be replicated in large mammals and humans.

Another practical limitation of small animal models is the size of their organs. The resolution at which spatial relationships like the spread of electrical potentials can be analyzed is severely constrained. This is especially relevant in the context of HFrEF, which is associated with many different forms of atrial and ventricular arrhythmias. It also makes a direct translation of interventional and surgical procedures into clinical practice impossible. Large animal models are therefore an important step toward accurately simulating human diseases and they have been and will continue to be invaluable in cardiovascular research.

There is an ongoing debate on which large animal model most closely resembles human cardiovascular physiology (5, 6). Historically, canines (dogs) were the preferred model organism, and many of the early landmark studies in heart disease, especially those concerning myocardial ischemia and arrhythmia, were conducted using this model (7, 8). An important drawback of the canine model is an extensive preformed epicardial collateral circulation, which can have a variable effect on infarct size.

The use of canine models has decreased over time, in part also due to cultural values. Animal experiments on dogs are often viewed unfavorably by the public when compared to other animal species. In some countries this has led to tougher regulations on canine experiments, which can constrain the study design and increase cost (9).

In recent decades, porcine (pig) models have become very popular for cardiovascular research. Their physiology, heart size, immune system and anatomy closely resemble that of humans and their coronaries have very little collateral circulation. It has been argued that in terms of coronary circulation, the pig heart therefore more closely resembles a “young heart,” while the dog heart with its extensive collaterals more closely resembles an “old heart” which has developed collaterals in response to chronic ischemic disease. However, this argument only extends to vascular anatomy and obviously not to the extensive remodeling that otherwise occurs in a human heart suffering from chronic ischemic disease.

A very important drawback of the pig model, is a high rate of sudden death due to tachyarrhythmias. A common approach to keep mortality at acceptable levels is the use of aggressive airway management, antiarrhythmic drugs and if necessary, defibrillation.

The ovine (sheep) model has many of the same attributes previously mentioned for pigs. There has been little effort to directly compare one against the other and they are both considered equally valid models. Other species that are rarely used include bovine (cow) and goat models.

**Figure 1:**
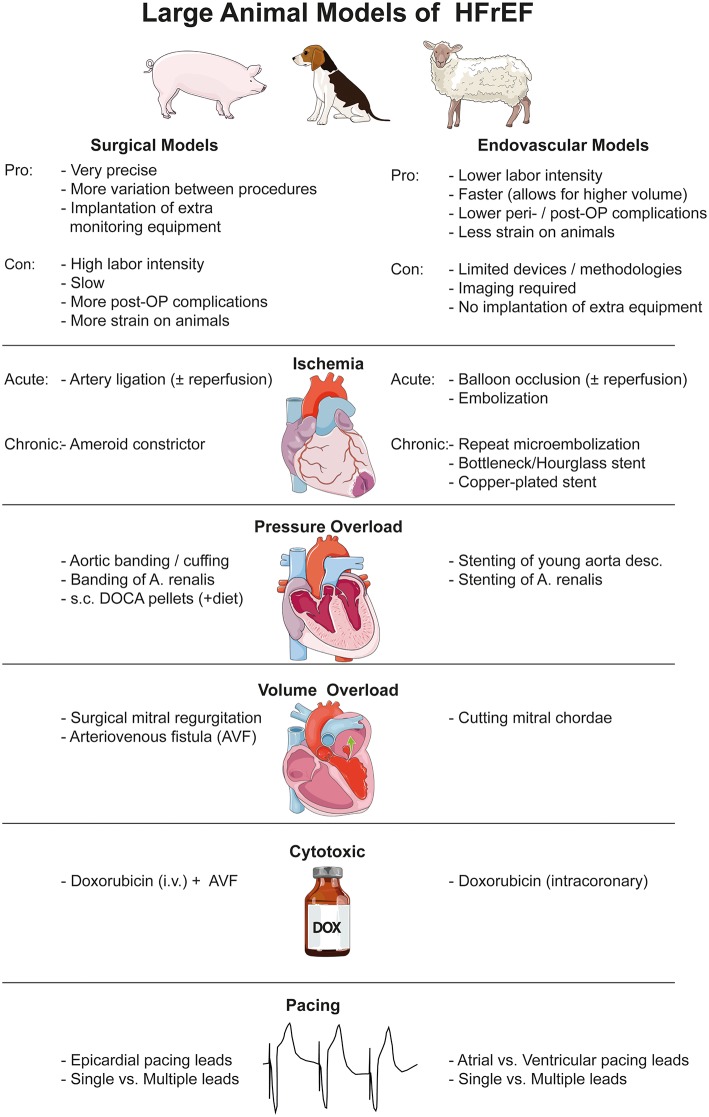
Graphical summary of large animal models of HFrEF, divided into surgical and endovascular methods. Images were obtained from https://smart.servier.com and are available under a creative commons license.

This review aims to give a short overview of the currently established large animal models of HFrEF. A short summary of the different models, their advantages and disadvantages as well as all references used in the main text are listed in Table 1. The literature on this topic is vast so we cite a few illustrative examples of each model, and refer them in Table 1. Figure 1 summarizes the pros and cons of surgical vs. endovascular models in general and briefly lists the different models.

**Table 1:** Overview of large animal models of HFrEF.

**Type**	**Method**	**Advantages**	**Disadvantages**	**Literature**
Acute ischemia	Embolization	Low-complexity, cheap	Less reproducible, less precise, no reperfusion possible	Canine (10) Porcine (11–13) Ovine (14, 15)
	LAD ligation	Reproducible, possibility of reperfusion, extra-coronary device implantation possible	Open-chest, lethal arrhythmias, surgical complications, labor intensive,	Canine (16) Porcine (17, 18) Ovine (19, 20)
	Balloon occlusion	Reproducible, possibility of reperfusion, closed-chest, fewer complications	Less precise than surgical ligation	Canine (21) Porcine (22–27) Ovine (28)
Chronic ischemia	Repeated microembolization	Good model of microvascular disease	Repeat interventions, labor intensive	Canine (29, 30) Ovine (31, 32) Bovine (33)
	Ameroid constrictor, bottleneck stent, copper-plated stent	Good model of single vessel disease	Variable degree of stenosis and LVEF reduction	Canine (34) Porcine (35–42) Ovine (43)
Pressure overload	Surgical aortic banding	Reproducible, minimal surgical procedure	Abrupt increase in pressure	Canine (44) Porcine (45, 46) Ovine (47)
	Stenting of descending aorta	Good model of aortic stenosis, gradual pressure overload, very similar to human conditions	Long time is necessary to develop left ventricular hypertrophy and dilation	Porcine (48)
	Renal artery stenosis	Good model of severe systemic hypertension, low-complexity, single intervention	Hormonal dysregulation, difficult to control	Canine (49, 50) Porcine (51) Ovine (52)
	DOCA pellet s.c. implantation with high salt diet	Simple non-invasive method	Mild systemic hypertension, uncertain effect on left ventricular function	Porcine (53)
Volume overload	Mitral regurgitation	Good model of volume overload, reproducible	Technically difficult	Canine (54–57) Porcine (58) Ovine (59)
	Arteriovenous fistula		Low clinical relevance, not well-established in large animals	Goat (60) Ovine (61)
Cytotoxic	Anthracyclines	Good model of doxorubicin-induced heart failure, simple non-invasive method	Narrow clinical relevance, toxic substances, often only in combination with other methods	Canine (62, 63) Porcine (64) Ovine (65), Goat (60)
Pacing	Rapid pacing	Good model of tachycardic remodeling	Reversible HF, qualitatively different remodeling	Canine (66, 67) Porcine (68–72) Ovine (73)

*Summarizes the different kinds of HFrEF models, their main uses and their advantages and disadvantages. The selected papers are meant to serve as illustrative examples of the various models, they do not represent a comprehensive list of all studies employing the respective model. LAD, Left anterior descending; LVEF, Left ventricular ejection fraction; DOCA, deoxycorticosterone acetate; sc, subcutaneous; HF, Heart Failure*.

## Myocardial Ischemic Models of HFrEF

### Models of Acute Myocardial Ischemia

In essence, large animal models of acute ischemia seek to induce or simulate acute myocardial infarction (AMI), which is a sudden and extensive ischemic injury, usually through total occlusion of a coronary artery. The most straight-forward models use intracoronary injections of thrombogenic material to permanently occlude a coronary artery. Commonly used materials include microbeads, collagen, gelatin, coils or autologous platelet aggregates (10–15). These models have the advantage of being fairly easy to carry out and of simulating the thrombotic nature of AMI, but they offer less precise control of infarct size than other methods. Additionally, they do not reflect the typical clinical setting in which AMI patients undergo percutaneous coronary intervention (PCI) and subsequent revascularization. Therefore, this model is most appropriate in studying the general mechanisms of acute myocardial ischemia and the subset of AMI with no subsequent revascularization.

In ischemia/reperfusion (I/R) models, ischemia is induced by either endovascular occlusion (e.g., balloon inflation) or surgical ligation of a coronary artery (16, 17, 19, 20, 28). After a set amount of time, the artery is re-opened, leading to reperfusion. Endovascular (percutaneous) approaches have the benefit of having lower peri- and post-interventional complication rates and of being most similar to the clinical setting, while surgical approaches allow for the implantation of additional equipment, for instance radio-opaque markers used by Mukherjee et al. to monitor infarct expansion over time (18). The percutaneous I/R model is especially relevant because it closely models the common clinical scenario of ST-elevation myocardial infarction, in which a prolonged period of ischemia is followed by revascularization through PCI (21–27).

### Chronic Myocardial Ischemia Models

HFrEF can also develop gradually as a consequence of progressive coronary artery disease and microvascular occlusions. A common approach to model this etiology is the use of repeated microembolizations, often using microspheres (29–33). Standard protocols often use weekly injections of microspheres between 40 and 100 μm until a desired EF is reached (31). While this method offers a gradual decline in left ventricular function with a lower mortality rate than acute models of ischemia, it is also very labor intensive, and puts more strain on the animals, since multiple interventions are necessary within a short period of time.

Another possibility is the use of implanted devices that gradually constrict a coronary artery. A surgical option is the ameroid constrictor, which is wrapped around a coronary artery, and expands gradually as it absorbs water (34–37, 43). This is also often combined with ligating a distal part of the coronary artery.

Using an endovascular approach there are multiple options to induce chronic occlusion. For example, Von Degenfeld et al. used a ligature in the middle of a bare-metal stent prior to implantation to induce an hourglass-shaped stenosis after deployment (38). In a similar fashion, Rissanen et al. used heat-shrink PTFE tubes to deform the distal end of their stent to create a bottleneck shape and immediately reduce blood flow (39). These methods create an empty space between implanted devices and the vessel wall. In this space, thrombus builds up and gradually constricts the vessel, while under dual anti-platelet therapy (DAPT), the lumen remains open. Varying DAPT dosage, the time-point of total occlusion of the vessel can be varied between 1–4 weeks. These models have the benefit of requiring only a single, short and low complexity intervention, however the acute mortality rate can be high with up to 37% in pigs (39). Others have relied on neointimal hyperplasia following implantation of copper-plated stents (40, 41) or polymer-coated stents (42). In these models, the development of hemodynamically significant stenosis usually takes 3–6 weeks. However, the degree of stenosis and the mortality rate differ greatly between the studies. Ultimately, the choice of model depends on the preferred time course of the stenosis, acceptable mortality rates and expertise, as all of these methods require experience to give reproducible results.

## Pressure Overload Models of HFrEF

Despite the fact that pressure-overload induced ventricular dysfunction secondary to either aortic stenosis or hypertension are very common clinical entities, few large animal models exist. In pressure overload, a gradual increase in afterload results in compensatory left ventricular (LV) hypertrophy, fibrosis and diastolic dysfunction (i.e., reduced compliance). These compensatory mechanisms eventually give way to decompensation and LV dilatation. Surgical models usually rely on surgical banding of the ascending or descending aorta (44–47). These models can vary in their approach in that some rely on producing an immediate aortic stenosis, others use a gradually inflating cuff, while implanting devices in young animals the aortic stenosis develops gradually, as the animals grow. Since aortic valve stenosis in humans develops slowly, models that induce an increasing degree of stenosis over time are likely more accurate in simulating HFrEF secondary to aortic stenosis.

A recent endovascular model for gradual development of pressure overload involves stenting of the descending aorta of young pigs. The intraluminal diameter of the stent stays constant while the pigs grow, leading to gradual aortic stenosis, LV hypertrophy and fibrosis (48).

Other models have aimed to simulate systemic hypertension. Important examples are induction of renal artery stenosis (49, 50), which is most established in dogs but has also been used in pig and sheep (51, 52). An interesting porcine model is the subcutaneous implantation of deoxycorticosterone acetate (DOCA) pellets, an aldosterone analog, combined with a high-salt diet, inducing systemic hypertension over time (53).

## Volume Overload Models of HFrEF

The most common clinical causes for volume-overload-induced-HFrEF are aortic and mitral regurgitation. Aortic regurgitation, however, is difficult to induce experimentally in a reproducible fashion, therefore most animal models have focused on inducing mitral regurgitation (54–59). Typically, a catheter-based intervention is used in which the mitral chordae are cut. This technique has been successfully used in dogs, pigs and sheep.

While the creation of arteriovenous fistulas is a common volume overload model in small animals, it is rarely used in large animal models and usually only in combination with other methods (60, 61).

## Cytotoxic Models of HFrEF

Cytotoxic HFrEF is a disease entity with growing clinical importance, and is most frequently encountered after chemotherapy with anthracyclines (74). Consequently, only few cytotoxic models of HFrEF exist, most of them using anthracyclines in combination with a surgically created arteriovenous fistula to induce heart failure. Tessier et al. administered 1–2 mg/kg of doxorubicin intravenously 2x/week for 13 weeks in goats, whereas Toyoda et al. used weekly intracoronary infusions of 0.7 mg/kg of doxorubicin over 5 weeks in canines (60, 62–65), while our group has administered doxorubicin and its derivatives in human doses in pigs (under publication).

## Pacing Models of HFrEF

Another reliable method to induce heart failure of varying degrees is tachycardic pacing. In this method, a pacemaker is used to induce tachycardia of typically between 180–240 bpm. This leads to biventricular dilatation, increased wedge pressure and an increase in systemic vascular resistance within 2 to 3 weeks (66). Different iterations of this model vary in their placement of the pacing wires in the atria or ventricles, with some even placing multiple leads to test various stimulation patterns (66–69, 73). Tachycardic pacing has been successfully used in canine, porcine, and ovine models (70–72). This model is particularly useful in studying HFrEF secondary to prolonged tachycardia. The HFrEF resulting from tachycardic pacing also differs from the other described models in some important respects. While in typical HFrEF, the reduction in left ventricular function is accompanied by changes in LV mass and fibrosis, this is not the case for pacing-induced HFrEF (70, 71). It is thought that prolonged tachycardic pacing leads to an intrinsic loss of myocardial contractility, which is further supported by the observation that contractile reserve in response to inotropic drugs is severely reduced (75). This reduction in contractility is reversible after the end of tachycardic pacing, with an often complete normalization of heart function within days or weeks (67).

## Conclusion

The choice of an appropriate animal model is the foundation of any successful translational research endeavor. Large animal models more accurately reflect human physiology and anatomy and are therefore invaluable in generating and translating basic research insights into clinical practice.

Here we have outlined the established large animal models for HFrEF categorized by which mechanism of HFrEF development is being modeled. This being a mini-review, an in-depth analysis of the various models are described in other reviews (76–78).

Ultimately, the choice, of which model is the most appropriate, is determined by the specific research question. The main three species discussed here, namely canine, porcine and ovine are all considered equally valid for most HFrEF related research questions and practical concerns like experience, available facilities, funding, and local regulations are often the main determinants in choosing one over the other.

Animal models have steadily been gaining in sophistication over time, but there is no single model or methodology that can accurately reflect the entire complex reality of HFrEF observed in human patients. This is especially true regarding the chronic nature of HFrEF and the numerous comorbidities that often afflict elderly HFrEF patients. Another level of complexity is added when medications are considered. While many models do include necessary medications like anti-platelet therapy or anti-arrhythmic drugs to ensure the survival of the animals, there is no consensus on whether they should also receive standard heart failure medications that would better reflect the clinical setting.

Nevertheless, the wide variety of large animal models presented here are the best approximations to human HFrEF that are currently available and they will surely continue to improve our understanding and thereby help improve patient outcomes.

## Author Contributions

AS wrote the main body of the manuscript. DT, DL, KZ, JM-T, MR, NP, and CM helped with literature research and gave feedback regarding style. AG, JW, and MG helped structure and revise the manuscript.

### Conflict of Interest Statement

The authors declare that the research was conducted in the absence of any commercial or financial relationships that could be construed as a potential conflict of interest.
